# Barriers and facilitators faced by individuals with intellectual disabilities and/or autism when accessing primary healthcare

**DOI:** 10.12968/bjnn.2022.18.6.268

**Published:** 2022-12-21

**Authors:** Chloe Poole, James Hill, Joanna Harrison, Alison Doherty

**Affiliations:** Clinical Research Support Officer, East Lancashire Hospital Trust; Synthesis, Economic Evaluation and Decision Science (SEEDS) Group, University of Central Lancashire; Synthesis, Economic Evaluation and Decision Science (SEEDS) Group, University of Central Lancashire; NIHR ARC NWC MIDAS Theme Manager \ Research Fellow (Faculty of Health & Well-being

**Keywords:** Autism Spectrum Disorder, Primary Healthcare, Barriers & Facilitators, Commentary, Integrative Review

## Abstract

Individuals with intellectual disability and/or autism spectrum disorder experience healthcare inequalities and have more unmet healthcare needs than the general population. Despite this, there is a sparsity of literature exploring the views of individuals with autism spectrum disorder or intellectual disability. This article summarises and evaluates an integrated review that explores the barriers and facilitators those with intellectual disability and/or autism spectrum disorder face when accessing primary healthcare.

## Introduction

Autism spectrum disorder is diagnosed by the presence of persistent deficits in social communication and interaction, understanding relationships, restricted and repetitive behaviours, insistence on sameness and either hypo or hyperactive sensory issues (Mughal et al 2022). The prevalence of autism spectrum disorder has substantially increased over the past decade (Chiarotti and Venerosi, 2020), and is now estimated to be about 60 cases per 10,000 people (Salari et al 2022) On average, adults with autism spectrum disorder have worse health when compared to the general public, and report more unmet healthcare needs (Nicolaidis et al, 2012; Cashin et al, 2018). These healthcare needs of people with autism spectrum disorder are not just physical health needs, but may also be mental health needs as people with autism spectrum disorder are commonly diagnosed with intellectual (Underwood et al 2019).

Intellectual disability is characterised by deficits in adaptive and intellectual functioning and has an approximate prevalence of 30 cases per 1000 people (Olusanya et al 2020). It has been shown that adults with autism spectrum disorder and/or intellectual disability may experience poor access to healthcare services (Bradshaw et al, 2019; Calleja et al, 2020). To identify why this may be the case, Doherty et al (2020) conducted a systematic review to explore the reported barriers and facilitators when accessing healthcare of individuals with intellectual disabilities, autism or both, as well as their carers. This review was selected for commentary because of its methodological strengths in both data retrieval, study selection and methods of synthesis.

This commentary aims to critically appraise the methods used within the review by Doherty et al (2020) and expand upon the findings in the context of clinical practice.

## Methods

This integrative review used systematic review methodology and was reported using the Preferred Reporting Items for Systematic Reviews and Meta-Analyses (PRISMA) 2009 checklist and reporting standards (PROSPERO CRD42018103103). A comprehensive database search was carried out using Ovid MEDLINE, Embase, CINAHL and COCHRANE. To be included in this systematic review, studies had to be published between 2001 and August 2018, and had to involve participants aged 14 years or older who had a previous formal diagnosis of or self-identified as having an intellectual disability and/or autism spectrum disorder. Studies involving the family members, support workers, carers and healthcare workers of individuals with autism spectrum disorder and/or intellectual disability were also included. Studies conducted before 2001 were excluded because of a change in legislation that impacted individuals with autism spectrum disorder and intellectual disability, and no language limitations were imposed on the inclusion criteria. Studies were required to be from a primary healthcare setting in the UK, or other countries that followed similar structures, funding and resource services. Systematic reviews, book reviews, editorials, epidemiological studies and prevalence studies were excluded. A mixture of quantitative, qualitative, mixed methods and randomised controlled trials were included.

Abstract and title screening, and full paper screening were undertaken by two independent reviewers. Any citation queries were discussed, and a consensus reached. The Mixed Methods Analysis Tool (MMAT) (Hong QN et al, 2018) was used to conduct the studies’ critical appraisal, which was carried out by two independent researchers. For the data synthesis, the research team developed and designed an extraction tool that used thematic analyses. Two independent researchers developed the themes to be used, with a third researcher being used when needed.

## Results

A total of 39 979 studies were initially identified. Following the screening process, a total of 63 studies remained (53 were of a qualitative nature, five mixed methods, four quantitative and one randomised controlled trial). Out of the 63 studies, 24 of these were conducted between 2002 and 2010, and 39 between 2011 and 2018. A total of 33 studies were from the UK, 13 from the United States and 18 from Australia, all noted High-Income Countries (HICs) in the systematic review (Doherty et al, 2020). Of the included studies, 46 were rated as high quality (all critical appraisal criteria met) two were rated as good quality (75% of the critical appraisal criteria met) and seven were rated as satisfactory (50% of the critical appraisal criteria met). The remaining eight studies were rated to be poor or very poor. The paper did not provide any more information on this breakdown, or which studies were appraised into which ‘category’. Through the process of thematic analysis, six main themes, including barriers and facilitators to accessing and using healthcare were identified (Table 1).

The first of the six themes identified was training for healthcare providers, with time constraints, lack of resources and uncertainties over specialist help being the main barriers for healthcare organisations to undertake training. Despite healthcare workers across both primary and secondary care settings appearing to recognise that this is essential to prevent knowledge gaps and uncertainties, there is still a perceived lack of knowledge and training. One of the methods identified as a possible avenue to enhance this process is to incorporate people with autism spectrum disorder and intellectual disability into the training team.

The second theme to be identified was the knowledge and awareness of how to support people with autism spectrum disorder and intellectual disability, and, in particular, how to make reasonable adjustments. It was identified that a lack of knowledge on how to support people with autism spectrum disorder and intellectual disability can result in possible negative attitudes of healthcare workers towards this population. Conversely, a warm, friendly and caring attitude from a healthcare worker can help to facilitate people with autism spectrum disorder and intellectual disability to access healthcare and discuss sensitive healthcare concerns.

Communication, the third theme listed, was noted as a significant barrier and is closely associated with the first two themes. It was identified that poor communication between healthcare workers and people with autism spectrum disorder and intellectual disability can lead to a misdiagnosis and the poor management of medications. As a consequence, poor communication can cause primary healthcare providers to rely on support workers, carers and family members to facilitate effective communication, which can potentially prevent service users from taking control of their own needs and care and can potentially increase their anxiety.

Another barrier was that the information provided was difficult to understand or for some individuals was incomprehensible. Participants’ suggestions to facilitate this were sign language, easy-to-read information and virtual appointments. Ensuring that individuals are seen by the same healthcare professional at each visit was something that both healthcare workers and those with autism spectrum disorder and/or intellectual disability agreed would be of benefit.

The fourth theme to be identified among people with autism spectrum disorder and/or intellectual disability was fear and embarrassment, and included fear of being judged on lifestyle choices, a fear of medical instruments and healthcare professionals using them on study participants, a fear of blood tests and vaccinations, and a lack of understanding about screening procedures. Therefore, this theme is linked with a lack of training for healthcare professions (theme one) and a lack of knowledge and awareness of how to support and adjust for people with autism spectrum disorder and/or intellectual disability (theme two). Without reasonable adjustments being made, participants reported that the whole experience of accessing healthcare can be frightening and increases their anxiety. This was because of issues such as not being fully aware of what happens during procedures, and their sensitivity to the unknown smells and noises of clinical areas. Suggestions from study participants to facilitate these included the use of photos, videos, coloured pictures, symbols and demonstration dolls.

Theme five was a lack of involvement in healthcare decision-making, which is linked to the third theme (communication). Healthcare providers often rely on others to make choices for the individual, even when they can make these decisions themselves. Involvement in making their own decisions allows for a feeling of empowerment and a better understanding of their diagnosis and treatment plan. Participants suggested that sharing inter-agency information in a joined-up approach would be useful in facilitating this, as well as tailored, person-centred and flexible services.

The final theme noted was time. Study participants noted that long waiting times can possibly cause an increase in anxiety, and limited time with healthcare professionals during consultations and appointments results in inadequate communication. Additional time is often required for effective communication with people who have intellectual disabilities and/or autism spectrum disorder.

## Commentary

### Critical appraisal of systematic review methods

Using the Joanna Briggs Critical Appraisal Tool for Systematic reviews, 10 of the 11 criteria were found to be satisfactory (Aromataris et al, 2015). The only criterion that was not achieved was the lack of methods to reduce error in data extraction, as it was unclear if data extraction was carried out by two independent reviewers. Other limitations of this review include using the MMAT to score each study depending on how many criteria were met. However, the recommended protocol when using the MMAT advises not to calculate a score but examine each criterion in the context to the outcome being assessed and give a detailed explanation of the impact of this criterion (Hong QN et al, 2018). Furthermore, while the researchers provided a breakdown of the scoring system and how many studies fell into each of the ratings, they did not report in detail exactly which of the studies fell into each rating. Considering this, and that unsatisfactory studies were also included in the synthesis, it is difficult to distinguish the quality of evidence underpinning each theme. Therefore, this makes it difficult to give any methodological recommendations for future research. Despite these limitations, the authors of this commentary deemed that the review is of high quality and provides an accurate and comprehensive summary of the results of the available studies that address the question of interest.

### Application of the review findings to practice

Based on the six themes identified, training appeared not only as a theme, but linked in with three of the other themes. Studies have found that healthcare workers across countries (United Kingdom united states and Australia) and services feel the need for more training, specifically on the behaviours, mental health and communication needs of those with autism spectrum disorder and intellectual disability (Weise and Trollor, 2018; Ghaderi et al, 2019; Ocanto et al, 2020; Urbanowicz et al, 2020). Thus, this interlinked theme of training may be a possible vehicle for improving access to healthcare for people with autism spectrum disorder and intellectual disability, as well as improving their experience. This review identified Public and Patient Involvement (PPI) as a possible avenue to optimise on training, which has previously been shown to enhance the delivery of mental health education, research and training (Seeralan et al 2021,Ward et al 2022). In this case, it would mean the inclusion of the service users and healthcare professionals to develop an effective training programme. By doing this, it would maximise the potential to include relevant key points the service users themselves feel are most prominent. Through an effective training programme, clinicians improve their knowledge and awareness on the reasonable adjustments that are available for people with autism spectrum disorder and intellectual disability. The review highlighted that specific areas where awareness should be increased is communication and alternative communication strategies. The focus of this increased awareness should also discuss the need of ensuring people with autism spectrum disorder and intellectual disability are involved in healthcare decisions, and a non-judgemental approach to involvement should be applied. Alongside these training needs, the importance of continuity of care was identified by service users and healthcare workers. Ensuring that, where possible, the patient sees the same healthcare worker, not only reduces costs (Wang et al 2021) but improves patient outcomes (Pereira Gray et al, 2018).

### Future research

Future research should explore the efficacy of these approaches in improving access to healthcare for people with autism spectrum disorder and intellectual disability, examining which particular barriers and facilitators are the most important in enabling people with autism spectrum disorder and intellectual disability to gain access to health and social care. As highlighted in the limitations of this review, there is limited research from low-income countries, indicating a need for examination of the barriers and facilitators within this population. Additionally, as this systematic review is now nearly 2 years old, there is a requirement for this review to be updated.

### CPD reflective questions

1. What are the main limitations to the systematic review?
2. What factors should be considered when working with people with autism spectrum disorder and intellectual disability?
3. What are the main training needs for clinicians working with people with autism spectrum disorder and intellectual disability?

This report is independent research funded by the National Institute for Health Research Applied Research Collaboration North West Coast (ARC NWC). The views expressed in this publication are those of the author(s) and not necessarily those of the National Institute for Health Research, the NHS or the Department of Health and Social Care.

## Figures and Tables

**Table 1:** List of six themes reported and a summary of the barriers and facilitators

Theme	Barrier	Facilitator
**Training**	Insufficient training for healthcare providers on specialist help and communication methods.	Training for healthcare providers facilitated by people with autism spectrum disorder and intellectual disability.
**Knowledge and awareness**	Healthcare services having a lack of knowledge, understanding and awareness around autism spectrum disorder and intellectual disability.	Warm, friendly and caring attitudes of healthcare workers can facilitate people with autism spectrum disorder and intellectual disability to access healthcare and discuss sensitive healthcare concerns.
**Communication**	Lack of awareness about different communication needs and styles.	Use of alternative communication styles.
**Fear and embarrassment**	Insufficient understanding of needs and adjustments.	Reasonable adjustments can be made, for example, demonstration dolls, easy-read information, photos, coloured videos, pictures, models and symbols.
**Lack of involvement in healthcare decision making**	Lack of involvement of the individual. Often rely on input from family, carers and support workers	Joined up approach and tailored services are highly regarded
**Time**	Long waiting times and insufficient time spent with the individual	Additional time is required for effective communication with people who have intellectual disabilities and/or autism spectrum disorder

## Key Points

- A total of six themes were identified: training, knowledge and awareness, communication, fear and embarrassment, lack of involvement in healthcare decisions, and time.
- Training was interlinked with communication and knowledge and may be used to overcome barriers.
- Research supports the idea that additional training is needed for healthcare professionals around autism spectrum disorder and intellectual disabilities.

## References

[R1] Aromataris E, Fernandez R, Godfrey CM (2015). Summarizing systematic reviews: methodological development, conduct and reporting of an umbrella review approach. Int J Evid Based Healthc.

[R2] Bradshaw P, Pellicano E, van Driel M, Urbanowicz A (2019). How can we support the healthcare needs of autistic adults without intellectual disability?. Current Developmental Disorders Reports.

[R3] Calleja S, Islam F, Kingsley J, McDonald R (2020). Healthcare access for autistic adults: A systematic review. Medicine.

[R4] Cashin A, Buckley T, Trollor JN, Lennox N (2018). A scoping review of what is known of the physical health of adults with autism spectrum disorder. Journal of Intellectual Disabilities.

[R5] Chiarotti F, Venerosi A (2020). Epidemiology of autism spectrum disorders: A review of worldwide prevalence estimates since 2014. Brain Sciences.

[R6] Department for Health and Social Care (2019). ‘Right to be heard’: The government’s response to the consultation on learning disability and autism training for health and care staff.

[R7] Doherty AJ, Atherton H, Boland P, Hastings R, Hives L, Hood K, James-Jenkinson L, Leavey R, Randell E, Reed J (2020). Barriers and facilitators to primary health care for people with intellectual disabilities and/or autism: An integrative review. BJGP Open.

[R8] Ghaderi G, Ghaderi G, Watson S, Watson S (2019). “In medical school, you get far more training on medical stuff than developmental stuff”: Perspectives on ASD from ontario physicians. J Autism Dev Disord.

[R9] Higor L, Claire L, Maneesh K (2020). COVID-19 outbreak: Implications on healthcare operations. The TQM Journal.

[R10] Hong QN, Pluye P, Fàbregues S, Bartlett G, Boardman F, Cargo M, Dagenais P, Gagnon M-P, Griffiths F, Nicolau B, O’Cathain A (2018). Mixed Methods Appraisal Tool (MMAT), version.

[R11] Málaga I, Blanco-Lago R, Hedrera-Fernández A, Álvarez-Alvarez N, Oreña-Ansonera VA, Baeza-Velasco M (2019). Prevalence of autism spectrum disorders in USA, europe and spain: Coincidences and discrepancies. Medicina.

[R12] Mughal S, Faizy RM, Saadabadi A (2022). StatPearls.

[R13] Nicolaidis C, Raymaker D, McDonald K, Dern S, Boisclair WC, Ashkenazy E, Baggs A (2012). Comparison of healthcare experiences in autistic and non-autistic adults: A cross-sectional online survey facilitated by an academic-community partnership. J Gen Intern Med.

[R14] Ocanto R, Levi-Minzi MA, Chung J, Sheehan T, Padilla O, Brimlow D (2020). The development and implementation of a training program for pediatric dentistry residents working with patients diagnosed with ASD in a special needs dental clinic. Journal of Dental Education.

[R15] Olusanya BO, Wright SM, Nair MKC, Boo NY, Halpern R, Kuper H, Abubakar AA, Almasri NA, Arabloo J, Arora NK, Backhaus S, Global Research on Developmental Disabilities Collaborators (GRDDC) (2020). Global Burden of Childhood Epilepsy, Intellectual Disability, and Sensory Impairments. Pediatrics.

[R16] Pereira Gray DJ, Sidaway-Lee K, White E, Thorne A, Evans PH (2018). Continuity of care with doctors a matter of life and death? A systematic review of continuity of care and mortality. BMJ Open.

[R17] Salari N, Rasoulpoor S, Rasoulpoor S, Shohaimi S, Jafarpour S, Abdoli N, Khaledi-Paveh B, Mohammadi M (2022). The global prevalence of autism spectrum disorder: a comprehensive systematic review and meta-analysis. Ital J Pediatr.

[R18] Seeralan T, Härter M, Koschnitzke C, Scholl M, Kohlmann S, Lehmann M, Eisele M, Braunschneider LE, Marx G, Scherer M, Löwe B (2021). Patient involvement in developing a patient-targeted feedback intervention after depression screening in primary care within the randomized controlled trial GET.FEEDBACK.GP. Health Expect.

[R19] Urbanowicz A, Parkin T, van Dooren K, Girdler S, Ciccarelli M, Lennox N (2020). The experiences, views, and needs of health professionals who provide care to adults on the autism spectrum. Research and Practice in Intellectual and Developmental Disabilities.

[R20] Underwood JFG, Kendall KM, Berrett J, Lewis C, Anney R, van den Bree MBM, Hall J (2019). Autism spectrum disorder diagnosis in adults: phenotype and genotype findings from a clinically derived cohort. Br J Psychiatry.

[R21] Wang YC, Lee WY, Chou MY, Liang CK, Chen HF, Yeh SJ, Yaung CL, Tsai KT, Huang JJ, Wang C, Lin YT (2021). Cost and Effectiveness of Long-Term Care Following Integrated Discharge Planning: A Prospective Cohort Study. Healthcare (Basel).

[R22] Ward K, Stanyon M, Ryan K, Dave S (2022). Power, recovery and doing something worthwhile: A thematic analysis of expert patient perspectives in psychiatry education. Health Expect.

[R23] Weise J, Trollor JN (2018). Preparedness and training needs of an australian public mental health workforce in intellectual disability mental health. Journal of Intellectual & Developmental Disability.

